# Spatio-temporal relief from hypoxia and production of reactive oxygen species during bud burst in grapevine (*Vitis vinifera*)

**DOI:** 10.1093/aob/mcv123

**Published:** 2015-09-03

**Authors:** Karlia Meitha, Dennis Konnerup, Timothy D. Colmer, John A. Considine, Christine H. Foyer, Michael J. Considine

**Affiliations:** ^1^School of Plant Biology, and The Institute of Agriculture, The University of Western Australia, Crawley, WA, 6009 Australia,; ^2^Freshwater Biological Laboratory, Department of Biology, University of Copenhagen, Universitetsparken 4, 2100 Copenhagen, Denmark,; ^3^Centre for Plant Sciences, University of Leeds, Leeds, Yorkshire LS29JT, UK and; ^4^Department of Agriculture and Food Western Australia, South Perth, WA, 6151 Australia

**Keywords:** Bud burst, *Vitis vinifera*, grapevine, reactive oxygen species, ROS, superoxide, hypoxia, oxygen partial pressure, meristem, development, respiration, ecodormancy, quiescence

## Abstract

**Background and Aims** Plants regulate cellular oxygen partial pressures (*p*O_2_), together with reduction/oxidation (redox) state in order to manage rapid developmental transitions such as bud burst after a period of quiescence. However, our understanding of *p*O_2_ regulation in complex meristematic organs such as buds is incomplete and, in particular, lacks spatial resolution.

**Methods** The gradients in *p*O_2_ from the outer scales to the primary meristem complex were measured in grapevine (*Vitis vinifera*) buds, together with respiratory CO_2_ production rates and the accumulation of superoxide and hydrogen peroxide, from ecodormancy through the first 72 h preceding bud burst, triggered by the transition from low to ambient temperatures.

**Key Results** Steep internal *p*O_2_ gradients were measured in dormant buds with values as low as 2·5 kPa found in the core of the bud prior to bud burst. Respiratory CO_2_ production rates increased soon after the transition from low to ambient temperatures and the bud tissues gradually became oxygenated in a patterned process. Within 3 h of the transition to ambient temperatures, superoxide accumulation was observed in the cambial meristem, co-localizing with lignified cellulose associated with pro-vascular tissues. Thereafter, superoxide accumulated in other areas subtending the apical meristem complex, in the absence of significant hydrogen peroxide accumulation, except in the cambial meristem. By 72 h, the internal *p*O_2_ gradient showed a biphasic profile, where the minimum *p*O_2_ was external to the core of the bud complex.

**Conclusions** Spatial and temporal control of the tissue oxygen environment occurs within quiescent buds, and the transition from quiescence to bud burst is accompanied by a regulated relaxation of the hypoxic state and accumulation of reactive oxygen species within the developing cambium and vascular tissues of the heterotrophic grapevine buds.

## INTRODUCTION

The buds of perennial trees and vines comprise one or more embryonic shoots with multiple meristems of diverse organogenic states, enclosed in a protective shell of dense scales. Similar to germinating seeds, the transition from quiescence to metabolically active occurring during bud burst is rapid, and requires the re-structuring of intercellular communication, respiratory and biosynthetic metabolism, and cell division and expansion. The identity, pluripotency and fate of cells in the meristem is determined by spatial organization (Esau, 1977; van den Berg *et al*., 1995), which is compounded in the embryonic shoot. Hence, this transition requires intricate spatial and temporal coordination of intercellular signalling networks within and between the functional domains of each meristem.

Oxygen is an essential substrate and signal in all aerobic organisms. Plants regulate the availability of oxygen and its metabolism during key transitions, including the regulation of quiescence (Considine and Foyer, 2014). Within this context the cellular reduction/oxidation (redox) hub plays a key role (Gapper and Dolan, 2006; Considine and Foyer, 2014), and we suggest the partial pressure of oxygen (*p*O_2_) also plays an important role, as known in animals and other aerobic organisms (Brahimi-Horn *et al*., 2007). The complex roles of redox processes in seed germination (Diaz-Vivancos *et al*., 2013, and references therein) and the control of *p*O_2_ are far from understood (Bradford *et al*., 2008; Borisjuk and Rolletschek, 2009). Similarly, our current knowledge of redox and *p*O_2_ sensing and signalling during bud burst is limited, particularly in terms of the spatial resolution of oxygen dynamics. Animal stem cell models consider that the redox environment, together with hypoxia (low *p*O_2_), are central regulators of the stem cell niche, which are key to cell identity and the maintenance of quiescence and pluripotency (Mohyeldin *et al*., 2010; Wang *et al*., 2013). The quiescent centre of the root meristem resides in an oxidized niche (Jiang *et al*., 2003; Jiang and Feldman, 2005). It is probable that the organizing centre and stem cells of the shoot apical meristems have similar requirements (Reichheld *et al*., 2007; Considine and Foyer, 2014).

In plants, as in animals, intracellular redox signals govern the cell cycle (Colucci *et al*., 2002; Jiang *et al*., 2003; Rothstein and Lucchesi, 2005; Diaz-Vivancos *et al*., 2010). The local perception of *p*O_2_ in animals enables acclimation during developmental transitions, as well as mediating responses to various stress conditions and pathologies (Brahimi-Horn *et al*., 2007). Recent studies have increased understanding of the sensing and signalling of *p*O_2_ in plant oxygen-stress responses (Gibbs *et al*., 2011; Licausi *et al*., 2011). However, this type of regulation has scarcely been studied in developing systems other than seeds.

Regulation of respiration is central to the transition from quiescence to the metabolically active state. During seed germination or bud burst, respiration increases because of the requirement for oxidative phosphorylation and reducing power (Morohashi and Shimokoriyama, 1975; Hourmant and Pradet, 1981; Bewley, 1997). Studies on seeds have demonstrated a regulatory role of redox signalling during germination and clear spatial gradients that illustrate the function of reactive oxygen species (ROS) and low-molecular-weight antioxidants in cell division and expansion (Gidrol *et al*., 1994; Schopfer *et al*., 2001; Oracz *et al*., 2009; Kranner *et al*., 2010; Rewers and Sliwinska, 2014).

The transition to bud burst can be accelerated by numerous sub-lethal stresses, including transient inhibition of respiration, heat shock or hypoxia (Esashi and Nagao, 1973; Erez *et al*., 1980; Erez, 1987), as is also the case with seed germination (Roberts, 1962; Siegel *et al*., 1962, 1964; Chen, 1970; Al-Ani *et al*., 1985). ROS are proposed to be key signalling agents induced by respiratory inhibition, as they function both directly on the cell cycle and by modulating activities of plant growth regulators such as ethylene, abscisic acid and auxin (Ophir *et al*., 2009). This fits with earlier suggestions that repressed catalase activity (Shulman *et al*., 1983; Nir *et al*., 1986) and increased production of hydrogen peroxide stimulate bud burst in grapevine (Perez and Lira, 2005; Vergara *et al*., 2012*a*). Indirect evidence that dormant buds reside in an hypoxic state comes from analyses of gene expression. Transcripts encoding proteins involved in oxidative phosphorylation and the tricarboxylic acid (TCA) cycle are repressed in dormant buds while those encoding components involved in glycolysis, pyruvate metabolism, fermentation and redox networks are increased (Halaly *et al*., 2008; Ophir *et al*., 2009; Vergara *et al*., 2012*b*). Much of these data come from buds under stress conditions.

**Fig. 1.**
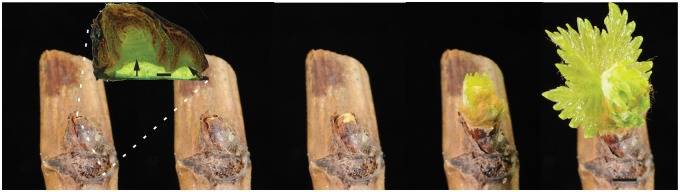
Time-series of grapevine bud burst. Single node explants of ecodormant buds were transferred from cool storage (4 °C) and planted out at 23 °C (dark). Figure shows the progression of bud burst at 0, 1, 3, 7 and 9 d (left to right) at 23 °C. Buds were sampled for the studies presented here at select time points during this development. The inset shows a sagittal section of the bud, with the primary (centre arrow), secondary (right arrow) and tertiary (left arrow) bud meristem complexes. When ecodormant (0 d), the bud complex is enclosed by a layer of lignified scales and several layers of bracts. Progressively over 3–5 d we observed expansion of the bud complex and rupture of the outer scales. Within 5–7 d, buds reached the stage of bud burst, according to the modified Eichorn–Lorenz scale (EL4; Coombe, 2004). By 9 d, the first leaves had separated from the shoot apical meristem (EL7). Scale bar in main figure = 5 mm, inset = 1 mm.

The scales of buds have low oxygen permeability and so the enclosed tissues are likely to be hypoxic, similar to the situation in dry seeds (Borisjuk and Rolletschek, 2009). In the seeds of some species, the suberized cell layers beneath the seed coat act as a barrier to oxygen diffusion, and their removal accelerates germination (Collis-George and Melville, 1974; Rolletschek *et al*., 2007). To date, no studies in the literature report data on *p*O_2_ values in buds. The following studies were therefore performed to resolve this issue, and to examine the cellular redox poise and *p*O_2_ status during bud burst. Furthermore, we aimed to resolve the spatio-temporal changes in these parameters that accompany the transition to bud burst, in a simplified developmental system that may provide a platform for further studies in a range of conditions and quiescent states (Considine and Foyer, 2014). The following experiments were performed on grapevine (*Vitis vinifera*), which is one of the most economically important woody perennial crop species, and has become a model species for research on perennial woody plants. Due to the anatomical complexity of the grapevine bud relative to other meristematic organs, it is useful to describe grapevine bud structure (Pratt, 1974; May, 2004). The mature bud complex, or N+2 according to May (2004), comprises a hierarchy of three buds – primary, secondary and tertiary – each resembling primordial shoots (Fig. 1). The primary bud is the most developed and by maturity bears 12–15 nodes, including inflorescence, tendril and leaf primordia, enclosed by layers of bracts and hairs. During maturation prior to winter, outer bracts lignify and harden to physically protect the bud over winter. Concurrent with this is a gradual cessation of meristematic activity and the acquisition of tolerance to desiccation and chilling (Schrader *et al*., 2004; Rohde *et al*., 2007; Ruttink *et al*., 2007). The cessation of growth involves the acquisition of dormancy, defined as the failure of an intact, viable bud to burst in otherwise conducive conditions, until repressive factors are overcome through entrainment to seasonal signals such as chilling and photoperiod (Bewley, 1997), otherwise known as endodormancy (Lang *et al*., 1987). Once endodormancy is overcome, the bud is said to be ecodormant, i.e. quiescent but awaiting conducive conditions for growth. In this study, we refer to the mature bud complex as a whole, although *p*O_2_ measurements were directed at the primary bud, and the secondary and tertiary buds were often lost during histological processing. The data presented here show that ecodormant buds undergo a regulated transition from hypoxia to the oxygenated state during bud burst. These findings provide a platform to further explore and dissect the roles of these signalling agents in mediating transitions in bud dormancy governed by environmental and developmental inputs.

## MATERIALS AND METHODS

### Plant material

Grapevine *Vitis vinifera* L.v ‘Crimson Seedless’ canes with mature dormant buds were harvested mid-winter from a vineyard in Yallingup Siding, Western Australia (33·694°S, 115·102°E). Canes with buds intact were stored at 4 °C in the dark until they had received at least 5500 chilling hours (approx. 7 months). The low degree of quiescence of the buds after cold-storage was confirmed by growing single-node cuttings of nodes 5–7 (explants, numbered acropetally) at 23 °C in vermiculite in darkness, with water maintained at field capacity (see Fig. 1 for developmental progression). Nodes 5–7 were chosen due to positional effects noted previously (Antcliff and May, 1961). The cumulative rate of bud burst was scored similarly to that described by Antcliff and May (1961) and according to the modified Eichorn–Lorenz scale (EL; Coombe, 2004), showing that 50 % of buds had reached EL-4 after 96 h at 23 °C and 80 % bud burst by 240 h (data not shown). On this basis we chose to study a time series over 72 h from transfer to 23 °C, in continuous darkness to minimize complexity. One or more single nodes were considered a biological replicate, as described for each assay.

### Internal O_2_ partial pressure

The internal *p*O_2_ of buds were measured after 3, 24 and 72 h at 23 °C, using a Clark-type oxygen microelectrode with tip diameter of 25 µm (OX-25; Unisense A/S, Aarhus, Denmark). Internal *p*O_2_ was also measured in buds with the outer scales removed by scalpel 10 min earlier, after 3 h at 23 °C. Microelectrodes were calibrated at atmospheric *p*O_2_ (20·87 kPa) and at zero O_2_, then mechanically guided into the buds, from the outer scale surface to the core of the primary bud, in 25 -µm steps to a depth of 2000 µm using a motorized micro-manipulator (MC-232; Unisense). The microelectrode recording was allowed to stabilize for 20 s after each step with measurements taken over the subsequent 10 s. Means and 95 % confidence intervals of individual buds (*n* = 3) were calculated using R (R Development Core Team, 2014) and graphics were compiled using the latticeExtra package and functions within (Sarkar and Andrews, 2013).

### Bud respiratory CO_2_ production

Four buds per biological replicate were excised from the cane by transverse sectioning at the base of the bud, weighed and placed onto thin agar plates, cut-side down, so that O_2_ entry and CO_2_ exit would occur across the bud scales rather than via the cut base. The rate of CO_2_ production of each biological replicate was measured in the dark, in an insect respiration chamber (6400-89; Li-COR, Lincoln, NB, USA) attached to an Li-6400XT portable gas exchange system. Measurements were performed at 23 °C, in CO_2_-controlled air (380 µmol CO_2 _mol^−1^ air) with 100 µmol m^−2 ^s^−1^ air flow, at 55–75 % relative humidity. The system was allowed to stabilize for 10 min before recording and until the ‘stableF’ value was equal to 1, i.e. the condition of humidity, CO_2_ and air flow were in equilibrium and stable. Means and 95 % confidence intervals were determined by fitting the time-series of CO_2_ evolution to a quadratic equation of the form, *y* = α + β_1_*x* + β_2_*x*^2^, using the linear model function within R (R Development Core Team, 2014) and plotted using ggplot2 (Wickham, 2009).

### Histology

Chemicals for histology were supplied by Sigma (St Louis, MO, USA) unless otherwise stated. To confirm the path of the *p*O_2_ microelectrode, buds were fixed for sectioning immediately after measurement. Before excision and fixation, a vector was cut in a sagittal plane from each side of the bud complex, adjacent to the primary bud and parallel to the path of the microelectrode to aid penetration of the fixative. Buds were then excised from the cane by transverse sectioning at the base of the bud, then fixed in 10 % (v/v) formaldehyde (Chem-Supply, Adelaide, Australia) with 5 % (v/v) propionic acid (Ajax Chemicals, Sydney, Australia) overnight at 4 °C, and subsequently dehydrated in serial ethanol solutions (15, 20, 25, 30, 50, 75, 90 and 100 %, v/v), 30 min each, with gentle agitation at 4 °C. Buds were then embedded in paraffin wax. Sagittal sections (5 µm) of the bud were made on a microtome (RM2255; Leica Biosystems, Nussloch, Germany), transferred to slides, de-waxed and stained with 0·05 % (w/v) toluidine blue O in 0·1 m phosphate buffer, pH 4·8. The sections were then scanned at 20× magnification using an Aperio Scanscope LX (Leica Biosystems).

Histological detection of hydrogen peroxide (H_2_O_2_) and superoxide (O_2_^.−^) were performed on bud sections from explants grown for 0, 3, 23 or 72 h at 23 °C. The methods of Groten *et al*. (2005) were followed with minor change: nitrobluetetrazolium (NBT) and 3,3′diaminobenzedine (DAB) were each dissolved in 10 mm phosphate buffer, pH 7·8, without dimethylsulfoxide. Buds were excised from the cane as described to visualize the path of the microelectrode, and stained under light vacuum for 8 h at room temperature in darkness. Stained buds were fixed in 4 % (v/v) formaldehyde (Chem-Supply) in a buffer of 5 mm MgSO_4_, 5 mm EGTA and 50 mm PIPES, pH 6·9, vacuum infiltrated for 1 h, incubated overnight at 4°C, dehydrated in serial ethanol solutions (15, 20, 25, 30, 50, 75, 90 and 100 %, v/v), 30 min each, with gentle agitation at 4 °C. The buds were then transferred to 1:1 (v/v) ethanol/Steedman’s wax solution (Norenburg and Barrett, 1987) and incubated overnight at room temperature prior to embedding. Serial sagittal sections of the bud were made at 20 -µm intervals using a microtome (RM2255; Leica Biosystems), transferred to slides and de-waxed in 100 % followed by 50 % (v/v), 5 min each solution. The sections were then scanned at 20× magnification using a Aperio Scanscope LX (Leica Biosystems).

To visualize lignin, NBT-stained buds were counter-stained with 0·05 % (w/v) Auramine-O (Ajax Chemicals) in deionized water. A drop of stain solution was placed on each section and left to absorb for 1 min before washing the slides with sprayed water. The stained sections were then visualized using a Carl Zeiss microscope (D-708 Z; Oberkochen, Germany) with blue light at 450–490 nm.

## RESULTS

### CO_2_ production and internal *p*O_2_

**Fig. 2.**
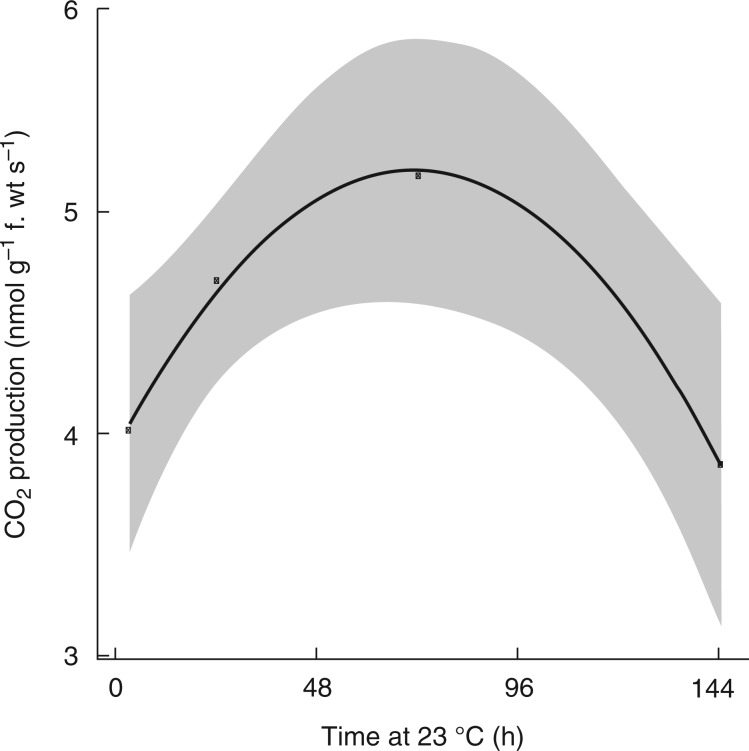
Respiratory CO_2_ production during grapevine bud burst. Ecodormant buds were transferred from cool storage (4 °C) and planted out at 23 °C (dark) at 0 h. The rate of CO_2_ production was measured on groups of four excised buds with the cut base on agar using an infra-red gas analyser in darkness. Data represent a regression (*n* = 4 replicates of four buds per replicate) ± 95 % confidence intervals by fitting the time series of CO_2_ evolution to a quadratic equation of the form, *y* = α + β_1 _*x* + β_2_*x*^2^ (refer Materials and Methods).

Respiratory CO_2_ production rates increased from approx. 4·0 to 5·2 nmolCO_2_ g f. wt^−1 ^s^−1^ in ecodormant buds maintained at 23 °C over the first 72 h following the transition from low to ambient temperatures. Subsequently, respiration rates fell to 4·0 nmol CO_2_ g f. wt^−1 ^s^−1^ by 144 h (Fig. 2), showing that metabolic activity was increased upon transition to conducive growth conditions for bud burst.

**Fig. 3.**
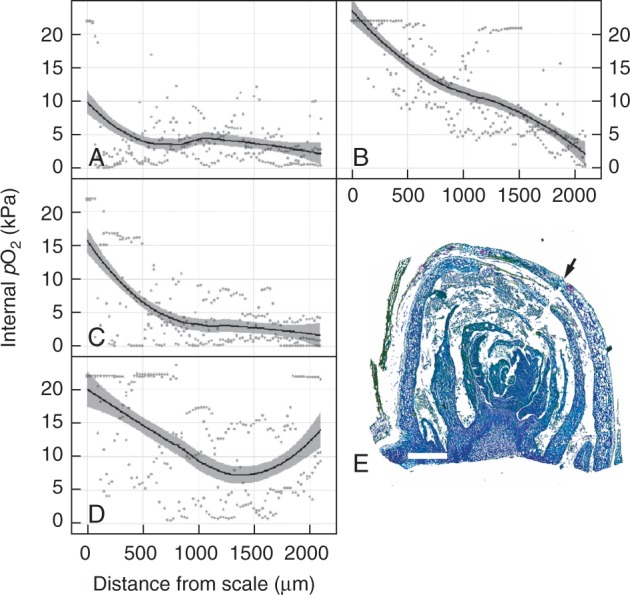
Internal profile of the partial pressure of oxygen (*p*O_2_) during grapevine bud burst. The *p*O_2_ of ecodormant buds, intact (A = 3 h, C = 24 h, D = 72 h) or with the outer scale removed (B = 3 h) was assayed after time at 23 °C in darkness. Data represent scatterplots of raw data (*n* = 3), with a regression curve applied and 95 % confidence intervals shown as grey shading. (E) Sagittal section of the primary bud meristem complex, fixed and stained with toluidine blue, showing the path of the O_2_ microelectrode from the outer scale (arrow) towards the inner core of the primary bud complex. Scale bar = 500 µm.

We determined the internal *p*O_2_ profile from the outer scale towards the core of the primary bud complex; at 3 h after transfer to 23 °C, which was the earliest stage of measurement, the internal *p*O_2_ was hypoxic immediately within the scale (approx. 10 kPa cf. air = 20·6 kPa), declined towards 5 kPa within the outer 500 µm and declined steadily to approx. 2·5 kPa through to the core of the bud complex (Fig. 3A). Some replicate data showed undetectable O_2_ (severe hypoxia/potential anoxia) at the core. Removal of the outer layer of scales at this time point resulted in oxygenation of the outer 15–1800 µm of the tissue profile, relative to the intact bud, although the core remained near 2·5 kPa (Fig. 3B). Despite this effect, de-scaling buds had no significant effect on the rate or completion of bud burst to stage EL-4, relative to intact buds (data not shown; see Materials and Methods). We then determined the *p*O_2_ profiles of intact buds at 24 and 72 h after transfer to 23 °C to determine whether removal of the scale at 3 h simply expedited the normal progression of oxygenation within the bud. By 24 h, only the *p*O_2_ of the outer 500 µm of the bud had increased, up to approx. 15 kPa *p*O_2_ immediately within the scale, while the remaining path towards the core remained near levels seen in intact buds at 3 h (Fig. 3C). By 72 h, the *p*O_2_ profile of the outer 1400 µm of tissue resembled that of the de-scaled buds at 3 h, although the *p*O_2_ of the inner 500 µm had increased, resulting in a biphasic profile such that the minimum *p*O_2_ along the electrode’s transect was approx. 7 kPa at 1400 µm depth from the scale, while at 2000 µm depth, the *p*O_2_ was >10 kPa (Fig. 3D). Figure 3E shows the path of the microelectrode in a representative section.

### Histological detection of superoxide and hydrogen peroxide

**Fig. 4.**
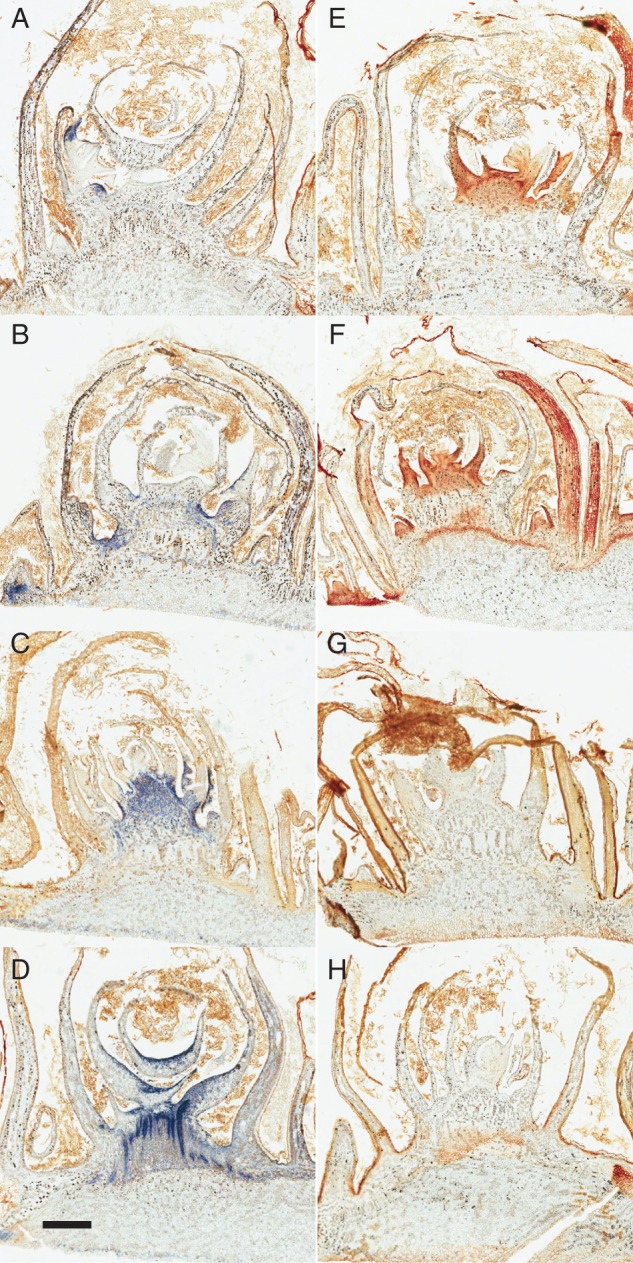
Spatial and temporal localization of reactive oxygen species (ROS) in sagittal sections of the primary bud meristem complex during bud burst. Superoxide (A–D) and hydrogen peroxide (E–H) localization were indicated using nitrobluetetrazolium (NBT) and 3,3′diaminobenzedine (DAB), respectively, against fixed sections (20 µm), sampled at 0 h (A, E), 3 h (B, F), 24 h (C, G) or 72 h (D, H) after transfer to 23 °C. Scale bar = 500 µm. Figures are representative of three independent replicates.

Using replicate buds of the same developmental series and treatment conditions as used for *p*O_2_ microelectrode measurements, we stained for the local accumulation of superoxide (O_2_^·−^) and hydrogen peroxide (H_2_O_2_), detected as the products of reactions with NBT or DAB, respectively. Immediately upon removal from 4 °C (0 h) and after 3 h at 23 °C, O_2_^.−^ accumulated in a very confined zone of the meristematic tissue, around the axillary meristems (Fig. 4A). After 3 h, however, O_2_^.−^ accumulation was observed in the cambial meristem tissues. For the first 3 h no H_2_O_2_ accumulation was detected in tissues around the apical meristem but low levels were observed in the cambial meristem tissue (Fig. 4E, F). After 24 h, O_2_^.−^ levels were increased in a wider zone of tissues of the apical meristem complex and retained in the cambial meristem tissues, while H_2_O_2_ was not accumulated in the tissues with the exception of the cambial meristem (Fig. 4C, G). At this time point the *p*O_2_ at the core of the bud complex remained low. A more distinct pattern of O_2_^·−^ localization emerged at 72 h, which suggested association with the developing pro-vascular tissues (Fig. 4D). At 72 h, no H_2_O_2_ accumulation was observed in the bud tissues (Fig. 4H). By this stage, the *p*O_2_ at the core of the bud complex had increased, suggesting a possible association between the patterns.

**Fig. 5.**
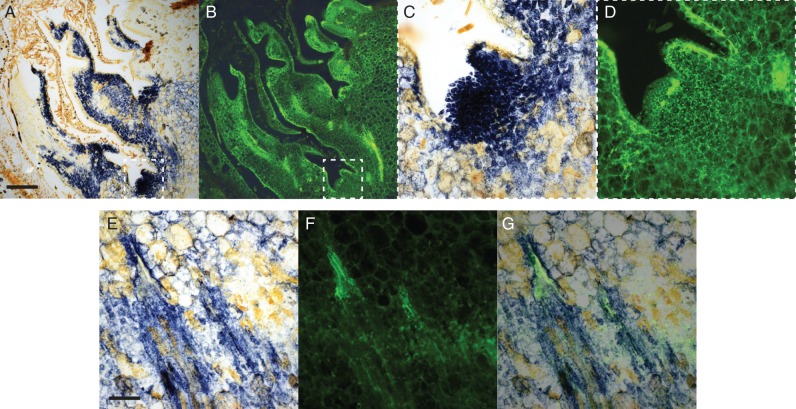
Spatial and temporal localization of superoxide (A, C, E) as contrasted to lignin (B, D, F) in grapevine buds during the first 3 h after transfer to 23 °C. Superoxide (NBT) is localized to latent meristem cells at 0 h, with negligible association with lignified cells (A–D, indicated by Auramine-O), where C and D are magnifications of the boxed inserts in A and B. By 3 h at 23 °C, superoxide production is evidently associated with lignin, indicative of pro-vascular development (E–G), where G is F superimposed over E. Scale bar = 100 µm (A, B), 20 µm (C, D), 50 µm (E–G). Figures are representative of three independent replicates.

To investigate the cell types associated with the distinct O_2_^.−^ pattern seen at 72 h, we counter-stained sections to visualize lignin. Figure 5 shows a clear co-localization of O_2_^·−^ with lignified cellulose as early as 3 h from transfer to 23 °C, but not earlier, providing further evidence that these are developing pro-vascular tissues. At 0 h, O_2_^·−^ accumulation was localized in the meristematic tissues but very little lignin associated with this pattern (Fig. 5C and D show magnified images of the boxed areas of Fig. 5A and B). By contrast, at 3 h the co-localization of O_2_^·−^ and lignin was observed (Fig. 5D–F shows the individual and superimposed images). Close inspection of Fig. 5E reveals the typical ladder-like perforation plates of xylem vessel elements.

## DISCUSSION

The experimental system presented here mitigated the potentially confounding effects of endodormancy and the influence of light. Endodormancy in grapevine, as in many perennial trees and vines, is primarily overcome by an accumulated exposure to chilling. Adequately chilled buds are termed ecodormant, a qualitative condition that is repressed only by the unfavourable growth environment (i.e. cold) and therefore more comparable to quiescence in other organs and forms of life. Bud burst per se does not require the presence of light (Pouget, 1963), although several studies have demonstrated influences of light intensity and photoperiod on organogenesis at other stages of development (Buttrose, 1970; Srinivasan and Mullins, 1981). There is no knowledge of whether photosynthesis may initiate in the bud prior to bud burst. Drawing analogy to seeds, where in several species photosynthesis influences the internal *p*O_2_ even during development or when mature and imbibed prior to germination (Borisjuk and Rolletschek, 2009), we may expect this to be the case in buds. Hence, overcoming endodormancy and excluding light allowed us to accurately and precisely study heterotrophic metabolism during the acute phase of bud burst.

Cells in a quiescent state are defined by very low metabolic rates, with minimal respiration until environmental or metabolic triggers prime the metabolic systems to resume growth. While several authors have described conserved responses to hypoxia or other oxidative stress across species and life forms (Hochachka, 1986; Jones *et al*., 2000; Mustroph *et al*., 2010), it is not possible to construct a generalized description of the metabolic state of quiescent cells or the changes that occur upon the transition to the metabolically active state or subsequent proliferation (Valcourt *et al*., 2012; Teslaa and Teitell, 2015). The findings of the present study provide new insights into the management of hypoxia when dormancy is broken in quiescent grapevine buds by exposure to chilling and the subsequent transition to ambient temperatures. While respiration rates are rapidly increased and superoxide accumulation is observed in and around the developing lignified zone of the cambium following the transition to ambient temperatures, the release from the hypoxic state is gradual and occurs in specific regions of the bud as the developmental transition progresses.

Rapid acceleration of respiratory CO_2_ production was observed in the buds following the transition from low to ambient temperatures, demonstrating alleviation of the constraints maintaining the quiescent state. This process, which was observed over the 72 h of bud burst measured at 23 °C, resembles the pattern observed during seed imbibition (Bewley, 1997) and in other studies on perennial buds (Hollis and Tepper, 1971; Shulman *et al*., 1983; Gardea *et al*., 1994; McPherson *et al*., 1997; Perez *et al*., 2008). Measurements of respiratory CO_2_ production do not allow discrimination between TCA cycle activity, fermentation, the pentose phosphate pathway or other pathways. Evidence suggests that fermentation occurs during bud burst under stress conditions and that the imposition of stress accelerates bud burst. For example, acetaldehyde and ethanol accumulate in ecodormant grape buds treated with sodium azide, hydrogen cyanamide or heat shock (Ophir *et al*., 2009). Hydrogen cyanamide, heat shock and hypoxia increase the levels of transcripts that are orthologues of *ALCOHOL DEHYDROGENASE*, *PYRUVATE DECARBOXYLASE* and *SUCROSE SYNTHASE* in ecodormant grapevine buds (Or *et al*., 2000; Ophir *et al*., 2009; Vergara *et al*., 2012*b*). However, in each case, untreated controls showed a slower or weaker transcriptional response with negligible fermentation activities observed during bud burst. These observations suggest that stress-induced changes in transcript profiles do not reflect the transcriptome signatures of developmental regulation of bud burst. Some evidence of pentose phosphate pathway activity was seen throughout seasonal development in pear buds (Zimmerman and Faust, 1969), and during chilling of potato tubers (Dwelle and Stallkneckt, 1978) or peony buds (Gai *et al*., 2013). However, these studies represent quite different physiological states compared with bud burst.

Many plant tissues and organs, including dry seeds, have permeability barriers that reduce oxygen diffusion. In the case of seeds, the hypoxic state may contribute to maintaining quiescence (see Introduction). The data presented here show that the scales of the dormant bud are a significant barrier to oxygen. Crucially, however, the meristematic core of the bud tissues remained in a hypoxic state even when the outer scales were removed. While Iwasaki and Weaver (1977) suggested some acceleration of bud burst in de-scaled ecodormant grapevine buds, removal of the outer scales did not affect the rate of bud burst in our study (data not shown). Schneider (1968) also showed that removal of scales attenuated quiescence of *Rhododendron* floral buds. However, in these earlier studies there was very limited replication of experiments. Nevertheless, it is conceivable that the buds used in our study were near to 100 % labile and hence very little effect of scale removal would be seen.

The data reported here demonstrate that the *p*O_2_ at the meristematic core of the bud complex was in an hypoxic state for up to 24 h after the environmental trigger to resume growth had caused an increased in respiration. Respiratory CO_2_ production rates had increased by 15 % in 24 h and superoxide accumulation was observed in the cambial tissues underlying the meristematic core of the bud complex. By 72 h, however, the oxygen profile was biphasic, the oxygen levels within the bud core had increased and superoxide accumulation was pronounced within the pro-vascular tissues. The present data are insufficient to explain the biphasic profile of oxygenation. In the heterotrophic conditions presented, even once the resistance to diffusion of the outer scales and compacted tissues was relaxed, the increased respiratory rates would contribute to substantial declines in *p*O_2_ with distance into the tissue. Further investigation of the vascular flow and metabolic activities at the core of the bud complex are required. Our group is currently exploring these features, and also the developmental processes and controls that preside in the presence of light, where photosynthesis may contribute to oxygenation even prior to bud burst, as is the case during germination of some seeds (Borisjuk and Rolletschek, 2009).

Vascular development and re-activation of intercellular communication are proposed to be essential early features of the transitions to and from quiescence in plant organs, including grapevine buds (Esau, 1948; Rinne *et al*., 2001; Paul *et al*., 2014). Cell expansion, cell-wall thickening and the conductivity of plasmodesmata in vascular tissues are all dependent on, or influenced by, ROS accumulation (Gapper and Dolan, 2006; Benitez-Alfonso *et al*., 2011). Ogawa *et al*. (1997) showed a strong co-localization of lignin and superoxide (NBT) in vascular tissue of spinach hypocotyls. Moreover, these authors demonstrated that inhibition of CuZn SUPEROXIDE DISMUTASE (CuZnSOD) or NAD(P)H OXIDASE reduced vascular lignin biosynthesis. More recently, ectopic expression of CuZnSOD and/or ASCORBATE PEROXIDASE (APX) in *Arabidopsis* resulted in enhanced vascular lignin synthesis (Shafi *et al*., 2015). SOD, APX and catalase were found in cell membranes that had been partially purified from lignin-producing tissues of Norway spruce (Karkonen *et al*., 2014). Together, these data suggest that vascular lignin synthesis is dependent on superoxide and/or hydrogen peroxide production. Note that hydrogen peroxide did not accumulate in vascular tissues of the buds studied here.

Taken together, the data presented here add to the growing body of evidence showing that regulation of redox and oxygen metabolism is critical to organ development (Considine and Foyer, 2014). The present study demonstrates that during bud burst, the complex network of enclosed shoot meristems undergoes a controlled transition from hypoxia to increasing *p*O_2_. This transition is accompanied by a highly localized accumulation of ROS in and around the developing cambium and vascular tissues. These data clearly demonstrate the spatial and temporal nature of the control of the oxygen and redox environments within the bud that occurs during the transition from quiescence to burst in heterotrophic grapevine buds.
